# Bimanual motor skill learning after stroke: Combining robotics and anodal tDCS over the undamaged hemisphere: An exploratory study

**DOI:** 10.3389/fneur.2022.882225

**Published:** 2022-08-18

**Authors:** Chloë De Laet, Benoît Herman, Audrey Riga, Benoît Bihin, Maxime Regnier, Maria Leeuwerck, Jean-Marc Raymackers, Yves Vandermeeren

**Affiliations:** ^1^Stroke Unit/NeuroModulation Unit (NeMU), Department of Neurology, CHU UCL Namur (Mont-Godinne), UCLouvain, Yvoir, Belgium; ^2^Louvain Bionics, UCLouvain, Louvain-la-Neuve, Belgium; ^3^Materials and Civil Engineering (iMMC), Institute of Mechanics, UCLouvain, Louvain-la-Neuve, Belgium; ^4^Clinical Division (NEUR), Institute of NeuroScience (IoNS), UCLouvain, Brussels, Belgium; ^5^Scientific Support Unit, CHU UCL Namur (Mont-Godinne), UCLouvain, Yvoir, Belgium; ^6^Department of Physical Medicine and Rehabilitation, CHU UCL Namur (Mont-Godinne), UCLouvain, Yvoir, Belgium; ^7^Department of Neurology and Neurosurgery, Clinique Saint-Pierre, Ottignies-Louvain-la-Neuve, Belgium

**Keywords:** stroke, motor skill learning, bimanual coordination, noninvasive brain stimulation (NIBS), robotics, primary motor cortex (M1), anodal tDCS

## Abstract

**Background:**

Since a stroke can impair bimanual activities, enhancing bimanual cooperation through motor skill learning may improve neurorehabilitation. Therefore, robotics and neuromodulation with transcranial direct current stimulation (tDCS) are promising approaches. To date, tDCS has failed to enhance bimanual motor control after stroke possibly because it was not integrating the hypothesis that the undamaged hemisphere becomes the major poststroke hub for bimanual control.

**Objective:**

We tested the following hypotheses: (I) In patients with chronic hemiparetic stroke training on a robotic device, anodal tDCS applied over the primary motor cortex of the undamaged hemisphere enhances bimanual motor skill learning compared to sham tDCS. (II) The severity of impairment correlates with the effect of tDCS on bimanual motor skill learning. (III) Bimanual motor skill learning is less efficient in patients than in healthy individuals (HI).

**Methods:**

A total of 17 patients with chronic hemiparetic stroke and 7 healthy individuals learned a complex bimanual cooperation skill on the REAplan^®^ neurorehabilitation robot. The bimanual speed/accuracy trade-off (biSAT), bimanual coordination (biCo), and bimanual force (biFOP) scores were computed for each performance. In patients, real/sham tDCS was applied in a crossover, randomized, double-blind approach.

**Results:**

Compared to sham, real tDCS did not enhance bimanual motor skill learning, retention, or generalization in patients, and no correlation with impairment was noted. The healthy individuals performed better than patients on bimanual motor skill learning, but generalization was similar in both groups.

**Conclusion:**

A short motor skill learning session with a robotic device resulted in the retention and generalization of a complex skill involving bimanual cooperation. The tDCS strategy that would best enhance bimanual motor skill learning after stroke remains unknown.

**Clinical trial registration:**

https://clinicaltrials.gov/ct2/show/NCT02308852, identifier: NCT02308852.

## Introduction

After a stroke, neurorehabilitation aims to restore patients' independence ideally through full recovery of impairments, activity limitations, and participation restrictions. A stroke involving the motor system may be particularly devastating, given that 30–66% of patients with stroke are left with chronic impairments and 40% do not recover independence in activities of daily life (1–3). Impairments of the upper limb, such as paresis, hypoesthesia, or spasticity, impede unimanual activities of daily life, which can be compensated by the adaptive recruitment of the nonparetic upper limb. By contrast, such strategic compensation cannot be used to rescue impaired bimanual activities of daily life (4). Indeed, a unilateral stroke specifically impairs bimanual motor control beyond its impact on the affected upper limb functions (5–7). This finding led to the development of specific scales quantifying limitations in bimanual activities, such as the ABILHAND scale (8) and that of bimanual neurorehabilitation programs (9–12).

Many actions involving both upper limbs are embedded as “default mode” patterns in the central nervous system, for example, the arms swing during walking. By contrast, most activities of daily life require asymmetrical, cooperative bimanual movements that have to be learned (13–15). During our whole life, we learn and refine bimanual motor skills such as opening a bottle, driving a car, or typing on a keyboard. Whether patients with hemiparetic stroke are able to achieve cooperative bimanual motor skill learning and how to enhance bimanual motor skill learning are key issues for developing efficient neurorehabilitation programs, which are not yet resolved. Furthermore, innovative interventions such as robotics and neuromodulation through noninvasive brain stimulation could help enhance bimanual motor skill learning.

Robotic devices allow control of many movement parameters, delivering high exercise doses, quantifying patient progress, manipulating feedback and reward, and interacting with patients through four main training modalities: active, active-assisted, passive, and resistive modes (16). Robotics exhibit great potential in neurorehabilitation, especially when combined with serious games and adaptive algorithms that monitor progression, dose rewards, and challenge patients without discouraging them (17, 18). Obviously, robotics is not a universal panacea, and whether and how robotic systems could be integrated within a comprehensive neurorehabilitation strategy remain to be tested.

Noninvasive brain stimulation, such as transcranial direct current stimulation (tDCS), can noninvasively modulate the activity of the healthy and damaged human brain and influence complex behaviors, such as learning (19, 20). The “historical” noninvasive brain stimulation model in stroke neurorehabilitation is based on the premise that a unilateral stroke induces an imbalance between interhemispheric interactions, which impedes the recovery potential of the damaged hemisphere (21). Accordingly, rebalancing dysfunctional interhemispheric interactions with noninvasive brain stimulation, either by restoring the excitability of the damaged hemisphere or by downregulating that of the undamaged hemisphere, would enhance recovery. Several randomized clinical trials based on this interhemispheric imbalance model yielded promising results. For example, combining tDCS with rehabilitation enhances motor recovery compared to sham tDCS in patients with stroke (22, 23). The mechanism by which tDCS specifically interacts with neurorehabilitation remains to be established. Possible mechanisms include diminishing fatigue, improving concentration or motivation, and/or enhancing motor skill learning (19, 24, 25). One of the key characteristics of motor skill learning is a shift of the speed/accuracy trade-off (SAT) (26), and we developed a serious game with motor skill learning based on this—the CIRCUIT (27, 28). More than an entertainment tool, the CIRCUIT was designed as a serious game for testing and inducing motor skill learning. Here, in patients with chronic hemiparetic stroke, we aimed to enhance motor skill learning on CIRCUIT through tDCS based on the interhemispheric imbalance model (25). Dual tDCS (i.e., anode over the primary motor cortex of the damaged hemisphere and cathode over M1 of the undamaged hemisphere) enhanced motor skill learning with the paretic upper limb compared to sham tDCS. Dual-tDCS led to an improved retention of the learned unimanual skill 1 week later, a generalization to a new version of the CIRCUIT, and improvements in other motor tasks, which were accompanied by consistent changes in activation and resting-state functional MRI patterns (29–31).

However, applying this dual-tDCS strategy to chronic hemiparetic stroke patients' learning, the *bimanual version* of the CIRCUIT task did not enhance motor skill learning compared to the sham (32). Clearly, the interhemispheric imbalance model does not account for the full spectrum of poststroke compensatory reorganization processes, calling for more comprehensive reorganization models (21, 24, 33–35); for example, according to the bimodal balance–recovery model, there is a subtle (and individual) interplay between interhemispheric competition and vicariation, depending on an individual threshold of structural reserve (i.e., the spared motor output of each hemisphere) (21). More recently, it has been suggested that tailoring the strategy of NIBS to individuals (based on the careful analysis of impairments and remaining motor pathways) could enhance its therapeutic effects (33–36). Recently, “excitatory” noninvasive brain stimulation with repetitive transcranial magnetic stimulation (rTMS) was shown to enhance bimanual force control in patients with hemiparetic stroke by targeting the undamaged (damaged) hemisphere in those with more (less) severe impairment (37). To date, tDCS studies inconsistently succeeded in enhancing bimanual motor control and/or motor skill learning in healthy individuals (38–43), and only a pilot study with five hemiparetic patients succeeded (44).

Therefore, given that (i) applying cathodal tDCS (supposed to be “inhibitory”) over the undamaged hemisphere can deteriorate the paretic upper limb function in severely impaired patients (45), (ii) the interhemispheric imbalance model does not integrate different poststroke reorganization mechanisms (21, 34, 46), (iii) the activity of both hemispheres during motor skill learning is actually *cooperative*, rather than competitive (47), (iv) bimanual cooperation requires a higher level of interhemispheric integration than unimanual movements (14, 48), and (v) the undamaged hemisphere might become the major hub sustaining bimanual coordination after a stroke (13), we tested a different tDCS strategy to enhance bimanual motor skill learning. We hypothesized that applying anodal tDCS over M1 of the undamaged hemisphere in the training of patients with chronic hemiparetic stroke on a neurorehabilitation robot enhances motor skill learning on the bimanual CIRCUIT task with superior retention 1 week later and increased generalization compared to sham tDCS. We further hypothesized that bimanual motor skill learning is less efficient in patients under sham tDCS than in healthy individuals. Finally, because the undamaged hemisphere could be more involved in bimanual cooperation in patients with more severe hemiparesis, we hypothesized that the severity of impairment correlates with the baseline bimanual performances and the impact of real compared to sham tDCS on bimanual motor skill learning.

## Methods

### Subjects

The research protocol was approved by the local ethics committee and complied with the Declaration of Helsinki. After providing written informed consent, 17 patients (Table 1, Supplementary Figures 1, 2) and seven healthy individuals were included. For patients, the inclusion criteria were patients (1) in the chronic stroke phase (> 6 months), (2) between 18 and 85 years of age, (3) having a motor deficit of the upper limb, and (4) having a stroke on brain imaging. Patients were excluded if they presented with (1) craniotomy/skull defect, (2) epilepsy, (3) intracranial metal, (4) inability to understand/execute commands, (5) drug/alcohol addiction, or (6) pregnancy. Neurological impairment was assessed using the upper limb Fugl-Meyer assessment (FMUL) and the National Institutes of Health Stroke Scale (NIHSS). Global activity limitation was evaluated with the modified Rankin Scale (mRS) and the ABILHAND questionnaire, and the dexterity of each hand was quantified with the box and blocks test (BBT) (8, 49). For healthy individuals, the inclusion/exclusion criteria were (1) 18–85 years, (2) no history of neurological disease, and (3) no intake of centrally acting drugs.

**Table 1:** Patients with stroke: Demographic data and baseline assessments.

	**Sex**	**Age (Year)**	**Time since stroke**	**Handed -ness**	**Damaged hemisphere**	**Location**	**NIHSS**	**FMUL**	**ABILHAND**	**ABILHAND (%logits)**	**BBT P**	**BBT NP**	**Composite**
1	F	28	>3 y	Right	Dom	cx	4	63	36	69.86	33.3	63.0	0.73
2	M	41	1–3 y	Right	N-Dom	cx	4	27	6	25.71	0.0	59.0	0.22
3	F	53	6–12 m	Right	Dom	sub-cx	4	58	36	70.00	68.0	76.7	0.82
4	F	48	>3 y	Right	Dom	sub-cx	2	65	39	84.00	75.3	77.6	0.93
5	M	64	6–12 m	Right	N-Dom	sub-cx	2	64	40	97.00	55.7	59.0	0.96
6	F	56	1–3 y	Right	Dom	sub-cx	0	62	37	77.00	44.0	45.3	0.89
7	M	71	>3 y	Right	Dom	cx	4	55	40	80.00	41.6	50.3	0.82
8	F	73	>3 y	Right	N- Dom	sub-cx	3	64	39	77.00	43.0	67.3	0.79
9	M	76	1–3 y	Right	N- Dom	cx	1	59	43	79.00	56.0	69.3	0.83
10	M	58	1–3 y	Right	N- Dom	sub-cx	1	58	41	74.00	46.3	58.0	0.81
11	F	67	1–3 y	Right	N- Dom	cx	2	65	40	80.00	52.0	56.3	0.90
12	M	75	>3 y	Right	N- Dom	cx	2	65	44	82.00	56.0	64.0	0.89
13	M	62	>3 y	Right	N- Dom	sub-cx	2	60	40	81.00	44.3	61.0	0.82
14	F	56	6–12 m	Right	Dom	cx	5	54	28	58.00	43.3	47.0	0.77
15	M	52	1–3 y	Right	N- Dom	sub-cx	4	26	7	30.72	2.6	66.0	0.25
16	F	75	6–12 m	Right	N- Dom	sub-cx	3	21	18	45.00	0.0	68.6	0.26
17	F	58	>3 y	Right	Dom	cx	2	59	39	84.27	41.3	63.3	0.80
Mean ± SD	9F/17	60			7Dom/ 17	8 cx/17	3	54.4	33.7	70.3	41.3	61.9	0.7
		± 13					± 1.4	± 14.6	± 13.8	± 19.5	± 21.9	± 9.1	± 0.2

*F, female; M, male; (N-)Dom, (non)dominant; cx, cortical; sub-cx, subcortical; NIHSS, National Institutes of Health Stroke Scale; FMUL, Fugl-Meyer Assessment for the Upper Limb; BBT, box and blocks test; P, paretic hand; NP, nonparetic hand*.

### Study design

**Figure 1:**
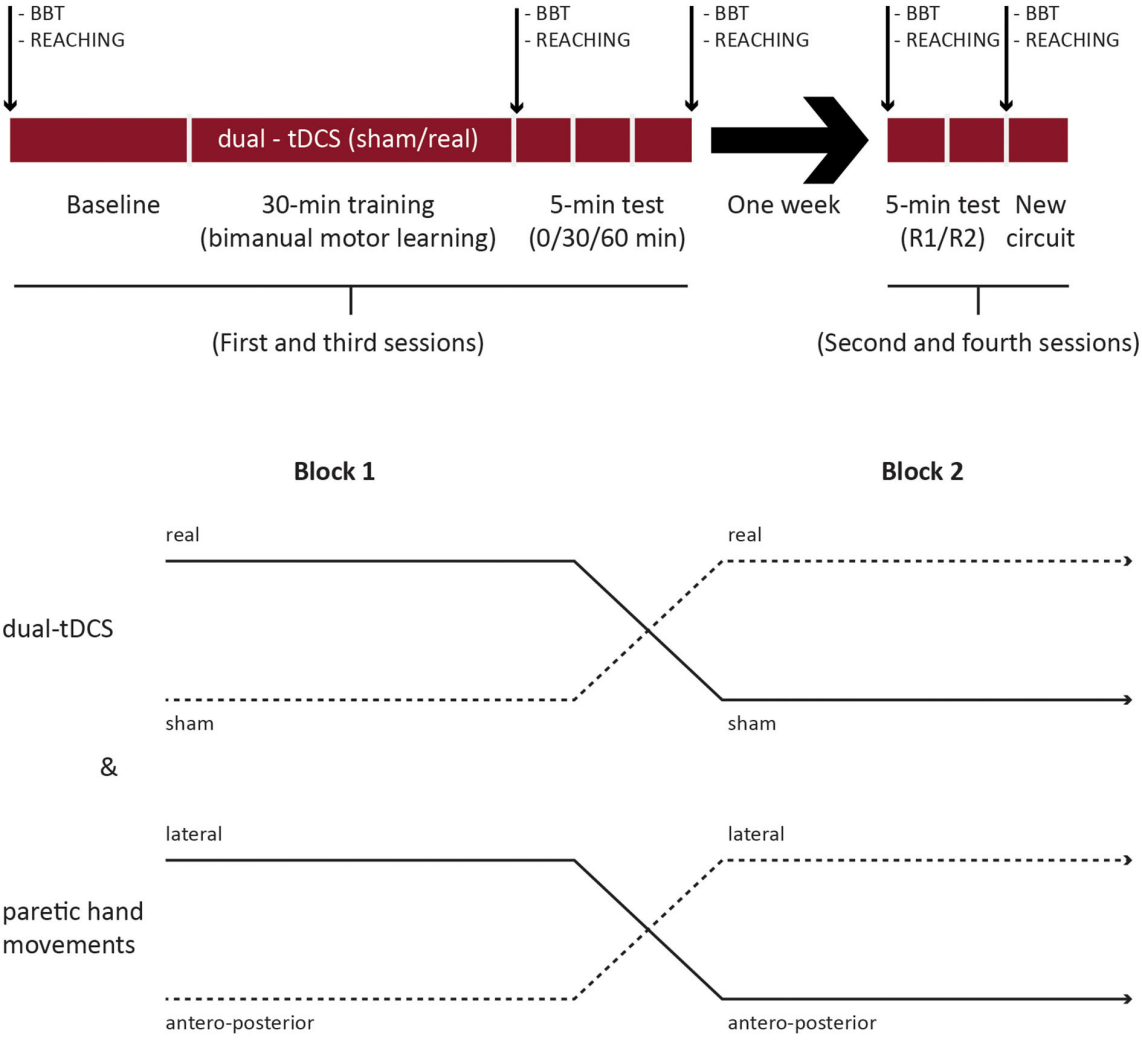
Study design. BBT, box and blocks test; R1/R2, retentions 1 and 2, respectively; new circuit: generalization.

The present study was a crossover, double-blind, and placebo-controlled randomized clinical trial. The first session included baseline evaluation, intervention (bimanual motor skill learning on the robotic device with CIRCUIT for 30 min under real/sham tDCS), and three evaluations over the 1st h of postintervention (Figure 1). After 1 week, during the second session, retention, and generalization were assessed. After a 1-week break, the intervention and retention sessions were repeated with the other tDCS condition and the other bimanual configuration (see later).

A total of three factors were randomized across patients and sessions: (1) tDCS condition (real/sham) and (2) bimanual configuration (i.e., the direction of movements controlled by the paretic upper limb: lateral/sagittal), and (3) CIRCUIT version [versions 1-2: same length and difficulty but different segment orders (27, 32)]. The randomization was performed by a third person using the online minimization software QMinim^®^^1^ (see Supplementary materials).

The healthy individuals performed only one intervention without tDCS and one retention session. Only the CIRCUIT version and the bimanual configuration (the direction of movements controlled by the nondominant upper limb) were randomized.

**Figure 2:**
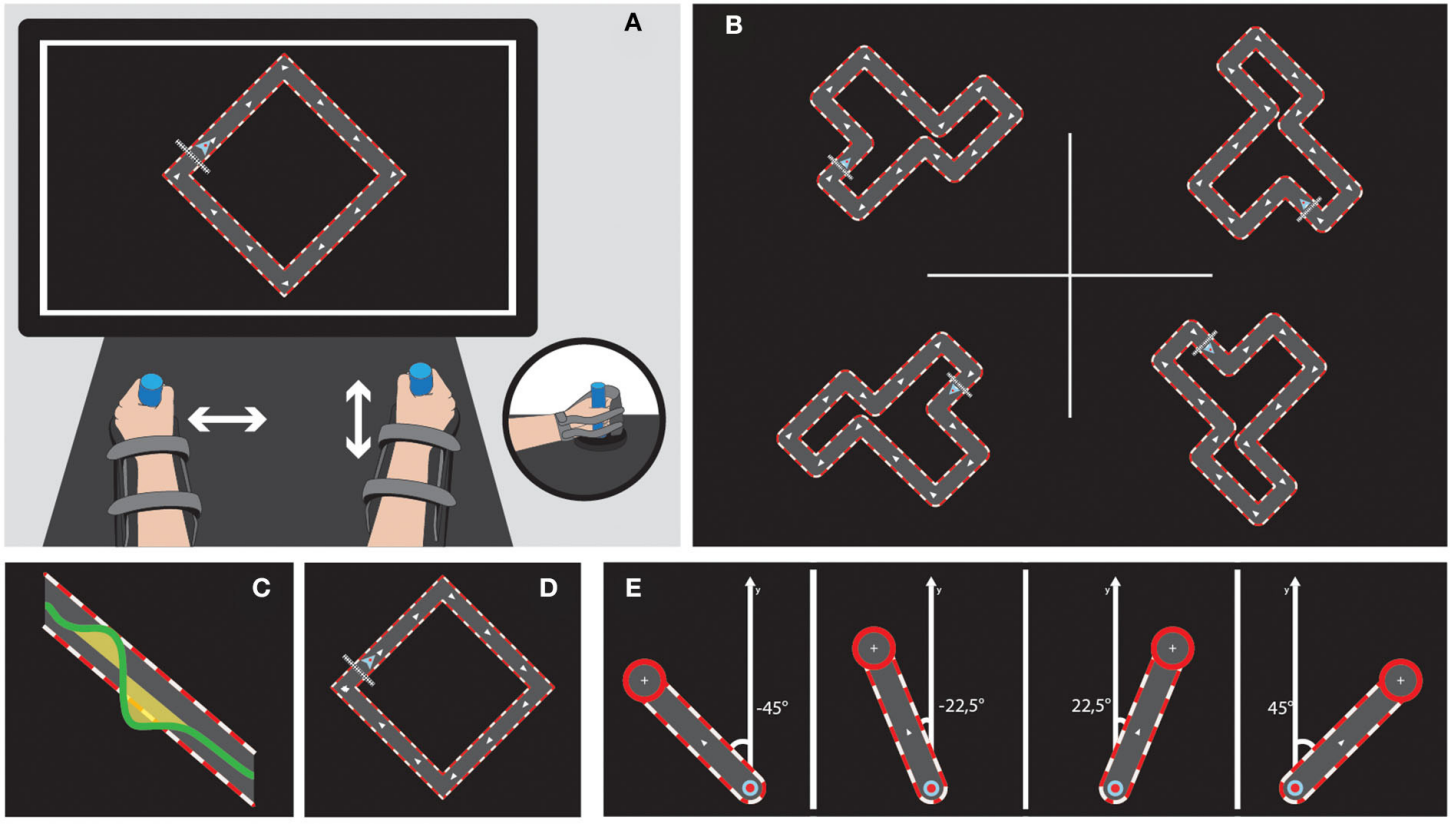
Bimanual tasks on the REAplan^®^. Upper left **(A)** General setup of the bimanual version of the REAplan^®^ robot. Note that each hand slides exclusively along one axis and thus controls a different direction of the common cursor (small arrowhead) displayed on the REAplan^®^ screen. The forearms rested in gutters and were strapped. Handles were adapted, if needed. Upper right **(B)** Four different circuits of identical length and difficulty. Bottom, from left to right: Cursor's displacement with regard to the ideal trajectory defined as the center of the circuit's track (surface = error) **(C)**, simple square used for familiarization **(D)**, and REACHING toward the four targets **(E)**.

In patients, the intervention session started with BBT testing for each hand (three trials of 60 s, nonparetic hand first). After tDCS electrode placement and short familiarization (see later), they performed 32 bimanual REACHING trials on the robotic device (Figures 1, 2). A baseline for the bimanual CIRCUIT was acquired, consisting of three 1-min blocks separated by 30 s of rest. Next, the patients trained on the bimanual CIRCUIT (1-min training block/30-s rest, 20 repetitions), and tDCS were started with the first block. Postintervention evaluations, identical to the baseline evaluation (three CIRCUIT blocks), were acquired immediately, as well as 30 and 60 min later. Finally, patients performed 32 bimanual REACHING trials and the BBT.

After 1 week, during the retention session, they performed two evaluations identical to baseline (three blocks each: R1 and R2) on the learned CIRCUIT with the same bimanual configuration. Generalization was assessed with (1) bimanual REACHING (32 trials), (2) a new version of the CIRCUIT both performed with the same bimanual configuration, and (3) the BBT.

The Fugl-Meyer assessments were performed by two trained physical therapists. The NIHSS and mRS were assessed by certified neurologists. The ABILHAND questionnaire and the box and blocks test were all conducted by the investigator CD. All operators were blinded to the allocation of the patients.

Some amendments were made to our historical trial protocol for the current exploratory study. In light of the previous results, the tDCS was adapted based on duration and electrode placement strategies. A longer stimulation of 30 min was proved to be efficient by a previous study (29). The electrode placement was designed to explore an alternative strategy after a failure of the “classical” strategy to enhance bimanual motor skill learning (32). As this was an exploratory approach, no fMRI was acquired in the current experiment. The safety and confidentially of patients were always maintained, and the amendments were approved by the local ethics committee.

### Bimanual tasks

The serious games CIRCUIT and REACHING were implemented on the bimanual version of the REAplan^®^ robotic system (AXINESIS, Wavre, Belgium), which has been successfully used after stroke (50). The subjects were comfortably seated in front of the REAplan^®^ screen. The subjects rested their forearms in gutters, and their hands grasped adjustable handles. Through virtual walls imposed by the REAplan^®^, one hand exclusively controlled lateral (left-right, X-axis) displacements of the common cursor displayed on the screen, and the other hand exclusively controlled sagittal (forward-backward, Y-axis) displacements (Figure 2). The cursor X-Y positions, velocities, and forces exerted against the virtual walls were sampled at 80 Hz and stored for offline analysis. After calibration by the REAplan^®^, bimanual familiarization was provided for 1 min with a simple square (Figure 2).

For bimanual CIRCUIT, the subjects were instructed to perform as many laps as possible with the common cursor during the 1-min blocks (speed constraint) while keeping the cursor within the track (accuracy constraint). The segments of the circuits were tilted 45° so that synchronized bimanual movements were required to displace the cursor perfectly within the track. To sustain motivation, an online countdown, the block's final score, and a high score were displayed.

For bimanual REACHING, the subjects were instructed to reach one of four targets appearing in a randomized order (eight trials/targets and a total of 32 trials). With respect to the midline (Y-axis), the targets were displayed at ±45° (symmetrical involvement of each hand, identical to the coordination needed for the CIRCUIT) or ±22.5° (asymmetrical hand involvement). After a wait time of 1 s, a target appeared with an ideal straight path from the home position. After reaching the target as fast and accurately as possible, the subjects had to maintain the position for 3 s on the target before returning to the home position.

### Transcranial direct current stimulation (tDCS)

The primary motor cortex (M1) of the undamaged hemisphere was localized by TMS using a figure-of-eight coil connected to a Magstim200^®^ (Magstim, Whitland, UK) to evoke reproducible movements in the nonparetic hand/wrist. Electrodes (35 cm^2^) were embedded in saline-soaked sponges. With an elastic band, the anode was secured over the M1 of the undamaged hemisphere hot spot, and the cathode was secured over the contralateral eyebrow after connection with an Eldith^®^ stimulator (NeuroConn^®^, Ilemenau, Germany). Eldith^®^ digit codes were used to deliver real/sham tDCS in a double-blind fashion. Under real conditions, after a fade-in of 8 s, stimulation was maintained at 1 mA for 30 min. Under sham conditions, stimulation faded out over 8 s.

### Data processing and outcome measures

For the bimanual CIRCUIT and REACHING serious games, the data were computed over 3-s bins that were averaged in 1-min blocks for statistical analyses and graphics. We computed three outcome measures (15, 32) with customized MATLAB^®^ routines (MATLAB 9.3 – R2017b, The MathWorks, Inc.) as follows.

1) Bimanual speed/accuracy trade-off (biSAT) in arbitrary units (a.u.) is given as follows:

$$\begin{array}{l} biSAT=\frac{velocity\;\left(\frac{cm}{s}\right)}{error\;\left(cm^{2}\right)}.C \end{array}$$

where C = 1 cm.s. Velocity is the first derivative of the cursor position. Error is quantified as the surface between the ideal path (defined as the center of the track) and the real trajectory of the cursor.

2) Bimanual coordination (biCO), in (a.u.):

$$\begin{array}{l} biCO=\frac{\min\left(\left|\frac{V_{x}}{\cos\propto }|,|\frac{V_{y}}{\sin\propto }\right|\right)}{\sqrt{{\left(\frac{V_{x}}{\cos\propto }\right)}^{2}+{\left(\frac{V_{y}}{\sin\propto }\right)}^{2}}} \end{array}$$

where α is the angle from the horizontal line and the line linking the start and arrival points.

3) Compound bimanual force exerted in the direction opposite to the axis imposed on each hand by the virtual walls (biFOP), in Newton (N):

$$\begin{array}{l} ||biFOP||=\;\sqrt{F_{y}^{2}+F_{x}^{2}} \end{array}$$

Given the natural propensity to perform identical/symmetrical movements during bimanual actions, biFOP should *decrease* (lesser forces exerted to no avail against the virtual walls) as biCO improves (i.e., increases).

To estimate the potential influence of the paretic upper limb motor function on bimanual motor skill learning, we computed an individual composite score as follows:


$$\begin{array}{l} Composite\;score\\ =\frac{\frac{FMUL}{66}+\frac{\%logits\;ABILHAND}{100}+\frac{BBT\;\left(paretic\right)}{BBT\;\left(non-paretic\right)}}{3} \end{array}$$

This composite score spans from 0 to 1, where values closer to 1 reflect a better overall residual motor function. For the FMUL, the individual scores were normalized by the maximal possible (i.e., normal) value. For ABILHAND, the individual %logits were divided by 100. For the BBT, we used the ratio between the scores of the paretic and nonparetic hands.

### Statistical analysis

*R* software (The R Foundation), including *nlme* and *ggplot2* packages, was used to generate generalized linear mixed models (GLMMs). For the primary outcome measures (biSAT, biCO, and biFOP), the data were presented on a logarithmic scale, and the GLMMs were performed on the log-transformed data. For the BBT, the raw data were used. The results are presented with 95% confidence intervals (CIs).

To screen for a potential carryover effect, descriptive analyses and estimates from GLMMs were performed on the baseline data with the tDCS conditions and bimanual configurations for biSAT, biCO, and biFOP. The patients were modeled as random effects, and [time], [time × stimulation order], [time × bimanual configuration], and [time × stimulation order × bimanual configuration] interactions were modeled as fixed effects.

The impact of real vs. sham tDCS on retention 1 week after intervention was computed using three GLMMs (one per outcome) comparing the first retention block (R1) with a baseline for biSAT, biCO, and biFOP.

The effects of real vs. sham tDCS during training were computed separately as the slopes of the changes over time (relative progression), which were subsequently compared. The patients were modeled as random effects, and [stimulation] and [stimulation × log2(bloc)] interactions were modeled as fixed effects.

Generalization was assessed with performances on the new CIRCUIT, REACHING, and BBT acquired 1 week after the allocated training. The effect of tDCS (real vs. sham) on biSAT, biCO, and biFOP was investigated by comparing the baseline to the new CIRCUIT (G). For bimanual REACHING, performances between baseline and retention were compared. For BBT, the numbers of blocks transferred by each hand were compared between baseline and retention.

Next, the baselines, progressions during training, retentions, and generalizations were compared between healthy individuals and patients under the sham condition using GLMMs modeled as described for the real vs sham analyses.

Finally, for the patients, Pearson's correlation coefficients between the composite score and (i) the baseline bimanual CIRCUIT performances and (ii) the impact of real vs. sham tDCS on bimanual motor skill learning were computed for biSAT, biCO, and biFOP.

## Results

### Bimanual motor skill learning

No carryover effects of the order of time, tDCS condition, bimanual configuration, or the [time × stimulation order × bimanual configuration] interaction were noted on the baseline data (Supplementary Figure 3). The patients learned and retained the bimanual skill under both tDCS conditions as measured for biCO and biSAT with the relative progression during training (Table 2) and retention 1 week after training, respectively (Supplementary Table 1). BiFOP did not show any significant improvement during training on CIRCUIT.

**Table 2:** Patients with stroke: Progression during training.

	**Outcome measures**	**tDCS**	**Relative progression [CI]**	***p***	**Difference of relative progression (real/sham) [CI]**	***p***
**Circuit**	biSAT	sham	1.15 [1.13–1.17]	<0.001		
		real	1.10 [1.08–1.12]	<0.001	0.96 [0.94–0.99]	0.0032
	biCO	sham	1.06 [1.05–1.08]	<0.001		
		real	1.05 [1.04–1.07]	<0.001	0.99 [0.97–1.01]	0.27
	biFOP	sham	1.03 [1.01–1.06]	0.017		
		real	1.00 [0.97–1.02]	0.79	0.96 [0.93–1.00]	0.06

*For CIRCUIT: Relative progression, slope of training-induced progression; Difference of relative progression, difference of relative progression between real and sham tDCS. tDCS, transcranial direct-current stimulation; CI, 95% confidence intervals; p, p-value, significance threshold at 0.05*.

**Figure 3:**
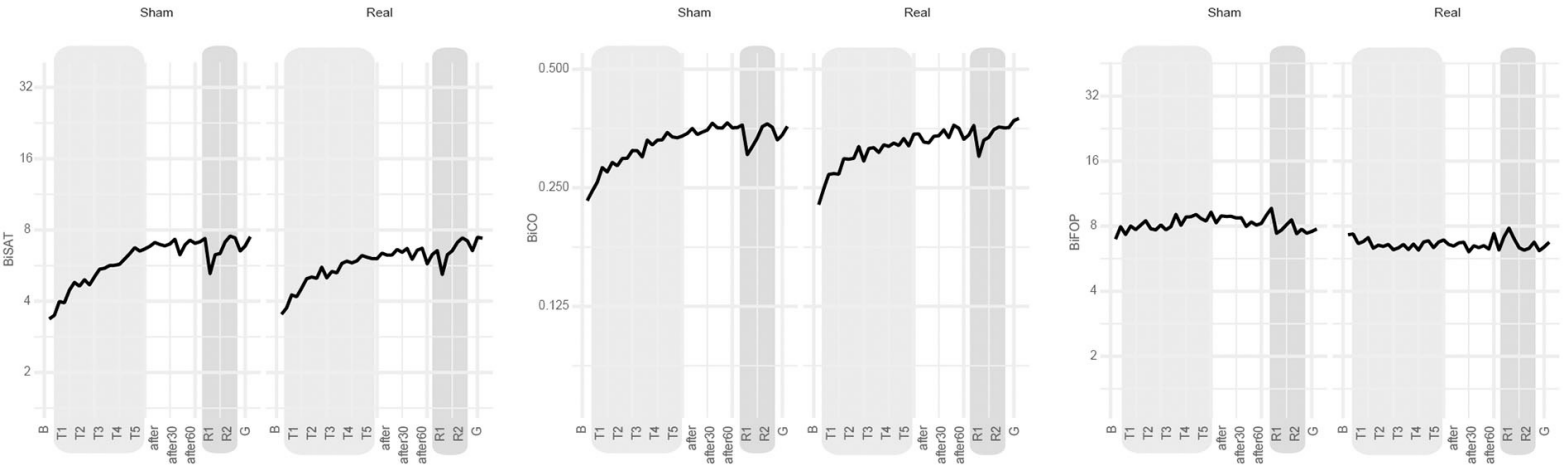
Main results. Evolution over time of biSAT (in a.u.), biCO (in a.u.) and biFOP (in N) on the bimanual CIRCUIT task at the group level (bold line: group's mean). B: baseline, T1-T5: bimanual training under sham or real tDCS, after-after30-after-60: evaluation immediately after intervention and 30 and 60 min later, respectively; R1-R2: retention blocks 1 and 2 at 1 week after intervention, respectively; G: generalization block (new CIRCUIT).

During training, the improvement on biSAT was slightly superior for sham (+15% [95% CI: 13 to 17%], Table 2) compared to real tDCS (+10% [8% to 12%], difference of relative progression: −4% [−6 to −1%] *p* = 0.0032, Table 2). At 1 week after intervention, no significant differences in the retention of the bimanual skill between real and sham tDCS were noted for biSAT, biCO, and biFOP (Table 3, Figure 3).

**Table 3:** Patients with stroke: raw data, retention, and generalization.

	**Outcome measures**	**tDCS**	**B**	**R1**	**R2**	**G**	**Retention**	**Diff. retention [CI]**	**p**	**Generalization**	**Diff. general. [CI]**	***p***
**Circuit**							**R1/B**	**Real/Sham**		**G/B**	**Real/Sham**	
	biSAT	sham	3.6	5.93	7.34	6.94	1.65			1.93		
		real	3.83	5.97	7.22	7.11	1.56	0.95 [0.83–1.08]	0.43	1.86	0.96 [0.84–1.10]	0.6
	biCO	sham	0.25	0.32	0.36	0.34	1.3			1.40		
		real	0.25	0.32	0.35	0.37	1.3	1.00 [0.92–1.08]	0.96	1.48	1.06 [0.97–1.15]	0.19
	biFOP	sham	7.4	7.73	7.87	7.57	1.04			1.02		
		real	7.09	7.04	6.41	6.43	0.99	0.95 [0.79–1.15]	0.59	0.91	0.89 [0.73–1.07]	0.21
**REACHING**										**G/B**	**Real/Sham**	
	biSAT	sham	11.13			24.93				2.24		
		real	12.23			27.75				2.27	1.01 [0.92 - 1.12]	0.80
	biCO	sham	0.24			0.32				1.33		
		real	0.24			0.33				1.35	1.02 [0.98–1.05]	0.38
	biFOP	sham	5.82			5.57				0.96		
		real	6.04			4.92				0.81	0.85 [0.76–0.95]	<0.001
**BBT**										**G–B**	**Real–Sham**	
	N-par	sham	65.8			68.0				2.2		
		real	63.1			68.0				5.0	2.7 [0.3–5.2]	0.028
	Paretic	sham	44.0			45.1				1.0		
		real	42.7			44.9				2.2	1.2 [−0.6–3.0]	0.18

*For CIRCUIT: B, baseline; R1 and R2: first and second retention blocks at 1 week after training; G, generalization (new CIRCUIT); Retention, retention from B to R1; Diff. Retention, difference in retention between real vs sham tDCS; Generalization, generalization from B to G to the new CIRCUIT; Diff. General., difference in generalization between real and sham. For REACHING and BBT, the testing performed at 1 week was placed in the G column. BBT, box and blocks test; N-par, nonparetic hand; Generalization, generalization from B to G measured on REACHING and BBT 1 week after training on CIRCUIT; Diff. General., difference in generalization between real and sham tDCS. tDCS, transcranial direct-current stimulation; CI, 95% confidence intervals; p, p-value, significance threshold at 0.05, biSAT, and biCO in arbitrary units (a.u.); biFOP in Newton*.

### Generalization

The patients generalized motor skill learning to a new CIRCUIT version (Supplementary Table 1), except for biFOP. No significant difference was noted between tDCS conditions for biSAT, biCO, and biFOP (Table 3, Figure 3). The patients also generalized performance improvement to bimanual REACHING (Supplementary Table 1). No significant differences were noted between tDCS conditions for biSAT and biCO, but biFOP improved more at 1 week after sham than at 1 week after real tDCS (ratio of relative progression real/sham: 0.85 [0.76 to 0.95], *p* < 0,001) (Table 3).

Comparing the unimanual BBT scores of baseline and retention for the paretic upper limb, no significant difference was noted between real and sham tDCS (+2.2 blocks vs. +1 block, difference: 1.2 blocks [−0.6 to 3], Table 3). For the nonparetic upper limb, performance improved more at 1 week after real tDCS than after sham (+5 blocks vs. +2.2 blocks, difference: 2.7 blocks [0.3 to 5.2], *p* = 0.028).

### Comparison with healthy individuals

The baseline performances were superior in healthy individuals compared to patients on bimanual CIRCUIT for biSAT (+65% [+21% to +126%], *p* = 0.0031) and biCO (+39% [+12% to +72%], *p* = 0.0041) but not for biFOP (Table 4, Supplementary Figure 5). The slope of training-induced improvement was steeper in healthy individuals than in patients for biSAT (+12% [7% to 17%], p < 0.001), but not for biCO and biFOP (Table 5).

**Table 4:** Healthy individuals and patients with stroke (sham): raw data, retention, and generalization.

	**Outcome measures**	**Group**	**B**	**R1**	**R2**	**G**	**Diff. Baseline [CI]**	***p***	**Retention**	**Diff. Retention [CI]**	**p**	**General**.	**Diff. General. [CI]**	***p***
**Circuit**							**HI/Stroke**		**R1/B**	**HI/Stroke**		**G/B**	**HI/Stroke**	
	biSAT	HI	5.95	11.91	16.27	15.57			2.00			2.61		
		stroke	3.60	5.93	7.34	6.94	1.65 [1.21–2.26]	0.0031	1.65	1.22 [1.04–1.42]	0.015	1.93	1.36 [1.16–1.59]	<0.001
	biCO	HI	0.34	0.44	0.45	0.46			1.28			1.36		
		stroke	0.25	0.32	0.36	0.34	1.39 [1.12–1.72]	0.0041	1.30	0.98 [0.89–1.08]	0.73	1.4	0.97 [0.89–1.07]	0.58
	biFOP	HI	7.49	8.80	8.85	7.61			1.17			1.02		
		stroke	7.40	7.73	7.87	7.57	1.01 [0.63–1.62]	0.96	1.04	1.12 [0.92–1.37]	0.25	1.02	0.99 [0.81–1.21]	0.94
**REACHING**												**G/B**	**HI/Stroke**	
	biSAT	HI	21.42			48.74						2.28		
		stroke	11.08			24.93						2.25	1.01 [0.89–1.15]	0.86
	biCO	HI	0.31			0.37						1.18		
		stroke	0.24			0.32						1.33	0.89 [0.85–0.93]	<0.0001
	biFOP	HI	6.15			6.37						1.04		
		stroke	5.75			5.57						0.97	1.07 [0.93–1.23]	0.33
**BBT**												**G-B**	**HI-Stroke**	
	HI	Dom	68.7			71.2						2.5		
	stroke	n-par	65.8			68.0						2.2	0.3 [−2.5–3.1]	0.84
	HI	n-dom	68.3			69.3						1.0		
	stroke	paretic	44.0			45.1						1.0	−0.02 [−2.2–2.1]	0.99

***HI**, healthy individuals; Stroke, patients with stroke (sham session). For CIRCUIT: **B**, baseline; **R1** and **R2**: first and second retention blocks at 1 week after training; **G**, generalization (new CIRCUIT); Diff. Baseline, difference at B between healthy individuals and patients with stroke; Retention, retention from B to R1; Diff. Retention, difference in retention between healthy individuals and patients with stroke; General., generalization from B to G to the new CIRCUIT; Diff. General., difference in generalization between healthy individuals and patients with stroke. For REACHING and BBT; the testing performed at 1 week was placed in the G column. BBT, box and blocks test; (n-)dom, (non)dominant hand; n-par, nonparetic hand; General., generalization from B to G measured on REACHING and BBT 1 week after training on CIRCUIT; Diff. General., difference in generalization between healthy individuals and patients with stroke. CI, 95%confidence intervals; **p**, p-value; significance threshold at 0.05; biSAT and biCO in arbitrary units (a.u.); biFOP in Newton*.

**Table 5:** Healthy individuals and patients with stroke (sham): Progression during training.

	**Outcome measures**	**Group**	**Relative progression [CI]**	**P**	**Diff. of Relative Progression (HI/Stroke) [CI]**	**p**
**Circuit**	biSAT	HI	1.23 [1.20–1.26]	<0.001		
		Stroke	1.15 [1.13–1.17]	<0.001	1.07 [1.04–1.10]	<0.0001
	biCO	HI	1.06 [1.05- 1.08]	<0.001		
		Stroke	1.06 [1.05–1.08]	<0.001	1.00 [0.98–1.02]	0.84
	biFOP	HI	0.99 [0.96–1.03]	0.73		
		Stroke	1.03 [1.01–1.06]	0.0032	0.96 [0.92–1.00]	0.06

*HI, healthy individuals; Stroke, patients with stroke (sham session). For CIRCUIT, Relative progression, slope of training-induced progression; Difference of relative progression, difference of relative progression between healthy individuals and patients with stroke. CI, 95% confidence intervals; p, p-value; significance threshold at 0.05*.

The retention after 1 week was greater in healthy individuals than in patients for biSAT (+22%, [+4 to +42%], *p* = 0.015), but not for biCO or biFOP (Table 4). Compared to the patients, the healthy individuals exhibited a similar generalization to the new CIRCUIT for biFOP and biCO but a better generalization for biSAT (relative difference: +36% [+16% to +59%], *p* < 0.001, Table 4). The patients also had a similar generalization to bimanual REACHING for biSAT and biFOP (Table 4) but had a smaller generalization than patients for biCO (−11% [−15 to −7%], *p* < 0.001).

For the BBT, the healthy individuals had a similar generalization to the patients for both the dominant/nonparetic hand and the nondominant/paretic hand (Table 4).

### Correlations with impairment

In patients, no significant correlation was noted between the composite score and the baseline CIRCUIT performance for biSAT, biCO, and biFOP (Table 6) or with the baseline REACHING performance (Table 6). At retention, no significant correlation was noted between the composite score and the impact of real vs. sham tDCS on bimanual motor skill learning for biSAT, biCO, and biFOP (Table 6).

**Table 6:** Correlations with the composite score in patients with stroke.

**Composite score vs**.	**Outcome measures**	**r [CI]**	***p*** **-value**
Circuit at baseline	biSAT	0.06 [−0.44–0.52]	0.83
	biCO	−0.04 [−0.51–0.45]	0.88
	biFOP	0.00 [−0.48–0.48]	0.99
Circuit Retention (Real vs. Sham)	biSAT	0.07 [−0.43–0.53]	0.80
	biCO	0.32 [−0.19–0.69]	0.21
	biFOP	−0.20 [−0.62–0.31]	0.44
REACHING at baseline	biSAT	0.01 [−0.47–0.49]	0.97
	biCO	−0.04 [−0.51–0.45]	0.87
	biFOP	−0.09 [−0.55–0.41]	0.73

*r, Pearson's correlations in patients between the composite score and different outcomes, CIRCUIT at baseline, baseline performance on the CIRCUIT; CIRCUIT retention (real vs. sham), ratio of real and sham retention performances on the CIRCUIT [(R1_Real_/B_Real_)/(R1_Sham_/B_Sham_)]; REACHING baseline, baseline performance on REACHING. CI, 95% confidence intervals; p, p-value; significance threshold at 0.05*.

## Discussion

Bimanual motor skill learning in patients with chronic hemiparetic stroke was not improved by anodal tDCS over the primary motor cortex (M1) of the undamaged hemisphere compared to sham tDCS. The healthy individuals exhibited higher baseline bimanual performances than the patients and achieved larger bimanual motor skill learning. Whereas real tDCS did not provide further enhancement compared to sham, bimanual motor skill learning on the neurorehabilitation robot REAplan^®^ resulted in the retention of a complex cooperation bimanual skill and generalization to untrained bimanual and unimanual tasks.

### Lack of tDCS-driven enhancement

Our previous studies in patients with chronic hemiparetic stroke were built on the interhemispheric imbalance model. Although dual-tDCS enhanced motor control with the paretic upper limb and motor skill learning on the unimanual CIRCUIT task (29–31, 51), it failed to enhance motor skill learning on the bimanual CIRCUIT version (32). Consequently, we hypothesized that anodal tDCS over M1 of the undamaged hemisphere could improve bimanual motor skill learning compared to sham tDCS. The main premises for this hypothesis were that the undamaged hemisphere may become the major hub for bimanual coordination after a stroke and that the activity of both hemispheres may actually be cooperative, rather than competitive, during motor skill learning (13, 47, 52, 53). However, anodal tDCS over M1 of the undamaged hemisphere also failed to enhance bimanual motor skill learning in patients with stroke. Interestingly, the BBT score of the nonparetic upper limb exhibited greater improvements 1 week after real vs. sham tDCS. This lasting enhancement of the nonparetic upper limb dexterity is consistent with a synergistic interaction between bimanual motor skill learning and anodal tDCS over M1 of the undamaged hemisphere.

Different noninvasive brain stimulation strategies, whether based on the interhemispheric imbalance model or on newer models, have consistently demonstrated their potential to enhance unilateral motor performances with the paretic upper limb (24, 54). By contrast, noninvasive brain stimulation approaches to enhance bimanual control/ motor skill learning in healthy individuals and patients with stroke have inconsistently succeeded to date (32, 38–44). One of the reasons might be that the effect size of tDCS is too inconsistent after stroke (55) or is too small compared to the large effect size of bimanual motor skill learning. Alternatively, the duration anodal stimulation may have been too long, leading to a reversion of the effect (56). More fundamentally, the generalizability of the interhemispheric imbalance model after stroke has been questioned, hinting at the need for a noninvasive brain stimulation approach stratifying individuals according to their reorganization processes (21, 24, 33–35). For example, to enhance recovery of the paretic upper limb, targeting the dorsal premotor cortex of the undamaged hemisphere with noninvasive brain stimulation, rather than M1 of the undamaged hemisphere, might be more efficient (34, 57). A recent study demonstrated that in patients with hemiparetic stroke performing flexion of both elbows, “excitatory” rTMS improved bilateral force coordination when applied over the dorsal premotor cortex of the undamaged hemisphere in more impaired patients and over M1 of the damaged hemisphere in less impaired patients (37). The neuronal mechanisms underlying tDCS and rTMS are different, with rTMS directly inducing cortical neuronal firing and tDCS modulating the resting neuronal membrane (20, 24). Despite these differences, in the context of neuromodulation and stroke rehabilitation, noninvasive brain stimulations (including rTMS and tDCS) are used primarily to increase/decrease and restore/interfere with cortical activity, with the aim of inducing behavioral enhancement (21, 24, 34). Thus, the optimal strategy to enhance bimanual motor skill learning with noninvasive brain stimulation, especially with tDCS, after stroke remains to be established.

### Comparisons with healthy individuals

The baseline performances on the CIRCUIT were superior in healthy individuals compared to patients for biSAT and biCO, as expected since bimanual coordination is impaired after stroke (4, 5, 13, 58, 59). By contrast, biFOP, which quantifies the amount of force “wasted” against the virtual walls, was not significantly different. This finding suggests that biSAT and biCO reflect overlapping bimanual cooperation processes, whereas biFOP captures another aspect of coordination (15), which is potentially less impaired in patients for this task. Alternatively, and perhaps more simply, since the task instructions emphasized SAT, rather than biFOP, the subjects might have simply not paid attention to the force applied on the walls.

Although both the healthy individuals and patients learned and retained, the healthy individuals overall performed slightly better. Generalization to a new CIRCUIT version did not differ between healthy individuals and patients. For bimanual REACHING, patients generalized slightly more than healthy individuals only on biCO, suggesting that patients had more room to improve bimanual coordination.

### Lack of correlation with impairment

During synergistically coupled limb movements, the undamaged hemisphere influences muscle activation of the paretic upper limb (4, 13, 58, 60). The undamaged hemisphere may become the major hub for bimanual cooperation after a stroke and play a key role in paretic upper limb recovery in severely impaired patients (13, 21, 34). Accordingly, we sought a correlation between impairment and the impact of tDCS on bimanual motor skill learning. However, the composite score did not exhibit a correlation. This finding might be due to the relatively low number of patients, a smaller effect size of tDCS compared to that of bimanual motor skill learning, or unidentified factors linked to the nature of the task. Nevertheless, the IC of those coefficients (ranging from −0.5 to 0.5) suggests that strong correlations are not to be expected. Furthermore, the relatively small confidence intervals suggest that the sample size was unlikely to drive this lack of effect and that increasing the number of patients would not result in a significant correlation (see Limitations).

### Bimanual neurorehabilitation and robotics

When training on the REAplan^®^ robot, the patients learned and retained the bimanual cooperation skill and generalized performance improvements in bimanual tasks (new CIRCUIT and REACHING) and unimanual gross dexterity (BBT). Furthermore, the robot and the bimanual tasks were truly well received by both patients and healthy individuals, who considered it more as a game than as a treatment. Since most activities of daily life are bimanual, such enhancements after a single session of bimanual motor skill learning are promising. Using (bimanual) robots opens new perspectives for delivering high-dose, high-intensity neurorehabilitation with virtually infinite possibilities for modulating the difficulty, feedback, and reward.

Interestingly, beyond learning how to coordinate the ULs, our cooperative bimanual motor skill learning likely involves a “technition” (or technical reasoning) component (61), that is, how to use a tool (here, the REA^2^plan). To accomplish the task (completing as many circuit laps as possible over 1 min), the subjects have to understand how to interact efficiently with the robot by establishing a control policy, that is, by mapping the sensory–motor mapping environment of the task. It has been proposed that a multidimensional cognitive mechanism could integrate action-related information by using semantic knowledge, sensorimotor knowledge, and technical reasoning (62–64). This concept is relevant for neurorehabilitation, to help patients recover how to use tools in everyday life.

### Limitations

The sample size was not large but was comparable to those of previous studies (29, 32, 37, 51, 57, 65). However small, the cohort used in this exploratory study showed relatively small confidence intervals, pleading for a robust interpretation of the outcomes. This approach implies a nuanced message: while we can exclude large and medium effects, we cannot exclude the presence of (very) small effects (66, 67). However, such small effects would lose clinical relevance.

In the current study, we did not use MRI or diffusion tensor imaging to quantify the amount of damaged/spared motor pathways. Modeling brain lesions and spared motor pathways might be a way to tailor tDCS and enhance its effect (68, 69). The current study was exploratory, and since we did not use a “focal” tDCS montage and used a functional approach to target M1 with TMS, we do not expect a significant added value of brain imaging at this point.

Crucially, before launching larger randomized clinical trials, it should be established whether tDCS could enhance bimanual motor skill learning after stroke, and the optimal strategy should be identified. Stratifying patients based on different poststroke reorganization processes might be a promising approach. It would be interesting to extend the bimanual robotic training over several days and to integrate it with physical/occupational therapy.

## Conclusion

In a previous study and in the current (follow-up) one, we explored two tDCS strategies to enhance bimanual motor skill learning in patients with chronic hemiparetic stroke. The first strategy, dual-tDCS, was based on the interhemispheric imbalance model. Although the strategy was successful for unimanual motor skill learning with the paretic upper limb (29–31, 51), it failed to enhance bimanual motor skill learning compared to sham tDCS (32). In the current experiment, based on the alternative hypothesis that the undamaged hemisphere became a compulsory hub for bimanual coordination/motor skill learning, we applied anodal tDCS over M1 of the undamaged hemisphere. This second strategy also failed, but again, the patients learned, retained, and generalized complex bimanual cooperation skills after a single training session. The tDCS strategy that would best enhance bimanual motor skill learning after stroke is unknown. The tDCS strategy that would best enhance bimanual motor skill learning after stroke is unknown. Maybe stimulating other areas involved in bimanual motor control such as the supplementary motor area or posterior parietal cortex could be efficient (14, 70). Nevertheless, bimanual cooperation motor skill learning with serious games implemented in robotic devices is promising for neurorehabilitation.

## Data availability statement

The raw data supporting the conclusions of this article will be made available by the authors, without undue reservation.

## Ethics statement

The studies involving human participants were reviewed and approved by Comité d'Ethique Médicale du CHU UCL Namur (Mont-Godinne). The patients/participants provided their written informed consent to participate in this study.

## Author contributions

YV and CD designed and conducted the study, including patient recruitment, data collection, data analysis with important input from AR, and prepared the manuscript draft with consistent intellectual input from all coauthors had complete access to the study data. BH supported the data collection on the robotic device, data processing, and the data analysis. BB and MR provided statistical support in analyzing the data and interpreting the statistical results. ML and J-MR were involved in patient recruitment. ML was involved in the clinical motor assessment of the patients. All authors contributed to manuscript revision, read, and approved the submitted version.

## Funding

The work of YV was supported by the following grants: Fonds de la Recherche Scientifique Médicale (FRSM) 3.4.525.08.F, Fonds Spécial de Recherche (FSR) from the UCLouvain, Fondation Van Goethem-Brichant, and Fondation Mont-Godinne. The work of Maral Yeganeh-Doost was supported by the following grants: FNRS-FRIA n° F3/5/5-MCF/ROI/BC-19727 and F3/5/5-MCF/XH/FC-17514 and Fondation Mont-Godinne 2018. The work of AR was supported by grants from the Fondation Mont-Godinne 2015-2016, Fonds Spécial de Recherche (FSR) of the UCLouvain 2016-2018, and Fondation Roi Baudouin/Fonds Amélie 2018-2019.

## Conflict of interest

The authors declare that the research was conducted in the absence of any commercial or financial relationships that could be construed as a potential conflict of interest.

## Publisher's note

All claims expressed in this article are solely those of the authors and do not necessarily represent those of their affiliated organizations, or those of the publisher, the editors and the reviewers. Any product that may be evaluated in this article, or claim that may be made by its manufacturer, is not guaranteed or endorsed by the publisher.

## Footnotes

^1^ http://rct.mui.ac.ir/q/index.php

## Abbreviations

B: baseline (CIRCUIT)
BBT: box and blocks test
CI: confidence interval
biCO: bimanual coordination
biFOP: bimanual force
biSAT: bimanual SAT
G: generalization (new CIRCUIT)
HI: healthy individual
R1: retention 1 (CIRCUIT)
R2: retention 2 (CIRCUIT)
SAT: speed/accuracy trade-off.
