# Supplementary material for: Bimanual motor skill learning after stroke: Combining robotics and anodal tDCS over the undamaged hemisphere: An exploratory study
Source: Front Neurol. 2022 Aug 18;13:882225. doi: 10.3389/fneur.2022.882225 (PMC9433746; doi:10.3389/fneur.2022.882225)
Supplement: Supplementary file 1 [file Table_1.docx]

### Supplementary Table 1: Stroke patients, Retention and Generalization inferences

|  | Outcome measures | tDCS | Retention | Retention [CI] | p | General. | General. [CI] | p |
| --- | --- | --- | --- | --- | --- | --- | --- | --- |
| **CIRCUIT** |  |  | **R1/B** |  |  | **G/B** |  |  |
|  | biSAT | sham | 1.65 | [1.50 – 1.81] | <0.001 | 1.93 | [1.75 – 2.12] | <0.001 |
|  |  | real | 1.56 | [1.42 – 1.71] | <0.001 | 1.86 | [1.69 – 2.04] | <0.001 |
|  | biCO | sham | 1.3 | [1.23 – 1.38] | <0.001 | 1.40 | [1.32 – 1.49] | <0.001 |
|  |  | real | 1.3 | [1.22 – 1.38] | <0.001 | 1.48 | [1.39 – 1.57] | <0.001 |
|  | biFOP | sham | 1.04 | [0.91 – 1.19] | 0.52 | 1.02 | [0.90 – 1.17] | 0.73 |
|  |  | real | 0.99 | [0.87 – 1.13] | 0.91 | 0.91 | [0.79 – 1.04] | 0.15 |
| **REACHING** |  |  |  |  |  | **G/B** |  |  |
|  | biSAT | sham |  |  |  | 2.24 | [2.08 – 2.41] | <0.001 |
|  |  | real |  |  |  | 2.27 | [2.11 – 2.44] | <0.001 |
|  | biCO | sham |  |  |  | 1.33 | [1.30 – 1.37] | <0.001 |
|  |  | real |  |  |  | 1.35 | [1.32 – 1.39] | <0.001 |
|  | biFOP | sham |  |  |  | 0.96 | [0.89 – 1.04] | 0.28 |
|  |  | real |  |  |  | 0.81 | [0.75 – 0.88] | <0.001 |
| **BBT** |  |  |  |  |  | **G – B** |  |  |
|  | N-par | sham |  |  |  | 2.2 | [0.5 – 4.0] | 0.011 |
|  |  | real |  |  |  | 5.0 | [3.2 – 6.7] | <0.001 |
|  | paretic | sham |  |  |  | 1.0 | [-0.2 – 2.3] | 0.11 |
|  |  | real |  |  |  | 2.2 | [1.0 – 3.5] | <0.001 |

For CIRCUIT: **B**: Baseline, **R1**: first Retention block at one week after training, **G**: Generalization (new CIRCUIT), **Retention:** Retention from B to R1, **Generalization:** Generalization from B to G to the new CIRCUIT. For REACHING and BBT, the testing performed at one week was placed in the G column. **BBT**: Box & Blocks test, **N-par**: nonparetic hand, **Generalization:** Generalization from B to G measured on REACHING and BBT one week after training on CIRCUIT. **tDCS**: transcranial Direct-Current Stimulation, **CI**: 95% confidence intervals, **p**: p-value, significance threshold at 0.05, **biSAT**, & **biCO** in arbitrary units (a.u.), **biFOP** in Newton.

## Supplementary Table 2: Healthy individuals and stroke patients, Retention and Generalization inferences

|  | Outcome measures | Group | Retention | Retention [CI] | p | General. | General. [CI] | p |
| --- | --- | --- | --- | --- | --- | --- | --- | --- |
| **CIRCUIT** |  |  | **R1/B** |  |  | **G/B** |  |  |
|  | biSAT | HI | 2.00 | [1.75 – 2.28] | <0.001 | 2.61 | [2.29 – 2.68] | <0.001 |
|  |  | stroke | 1.65 | [1.51 – 1.79] | <0.001 | 1.93 | [1.77 – 2.09] | <0.001 |
|  | biCO | HI | 1.28 | [1.18 – 1.39] | <0.001 | 1.36 | [1.26 – 1.48] | <0.001 |
|  |  | stroke | 1.30 | [1.24 – 1.37] | <0.001 | 1.4 | [1.33 – 1.47] | <0.001 |
|  | biFOP | HI | 1.17 | [0.99 – 1.39] | 0.06 | 1.02 | [0.86 – 1.20] | 0.85 |
|  |  | stroke | 1.04 | [0.94 – 1.16] | 0.42 | 1.02 | [0.92 – 1.14] | 0.67 |
| **REACHING** |  |  |  |  |  | **G/B** |  |  |
|  | biSAT | HI |  |  |  | 2.28 | [2.04 – 2.54] | <0.001 |
|  |  | stroke |  |  |  | 2.25 | [2.09 – 2.42] | <0.001 |
|  | biCO | HI |  |  |  | 1.18 | [1.14 – 1.22] | <0.001 |
|  |  | stroke |  |  |  | 1.33 | [1.30 – 1.36] | <0.001 |
|  | biFOP | HI |  |  |  | 1.04 | [0.92 – 1.16] | 0.54 |
|  |  | stroke |  |  |  | 0.97 | [0.90 – 1.04] | 0.41 |
| **BBT** |  |  |  |  |  | **G-B** |  |  |
|  | HI | dom |  |  |  | 2.5 | [0.2 – 4.9] | 0.04 |
|  | stroke | n-par |  |  |  | 2.2 | [0.7 – 3.8] | 0.004 |
|  | HI | n-dom |  |  |  | 1.0 | [-0.8 – 2.8] | 0.27 |
|  | stroke | paretic |  |  |  | 1.0 | [-0.1 – 2.2] | 0.08 |

**HI:** Healthy individuals, **Stroke:** Stroke patients (Sham session). For CIRCUIT: **B**: Baseline, **R1:** first Retention block at one week after training, **G**: Generalization (new CIRCUIT), **Retention:** Retention from B to R1, **Diff. Retention:** Difference in retention between healthy individuals and stroke patients, **General.:** Generalization from B to G to the new CIRCUIT. For REACHING and BBT, the testing performed at one week was placed in the G column. **BBT**: Box & Blocks test, **(n-)dom:** (non)dominant hand, **n-par**: nonparetic hand, **General.:** Generalization from B to G measured on REACHING and BBT one week after training on CIRCUIT.**CI**: 95% confidence intervals, **p**: p-value, significance threshold at 0.05, **biSAT**, & **biCO** in arbitrary units (a.u.), **biFOP** in Newton.
