# Supplementary material for: Bimanual motor skill learning after stroke: Combining robotics and anodal tDCS over the undamaged hemisphere: An exploratory study
Source: Front Neurol. 2022 Aug 18;13:882225. doi: 10.3389/fneur.2022.882225 (PMC9433746; doi:10.3389/fneur.2022.882225)
Supplement: Supplementary file 2 [file Data_Sheet_1.docx]

# Supplementary Materials

## Supplementary Methods

Supplementary Figure 1. Simplified CONSORT flow diagram


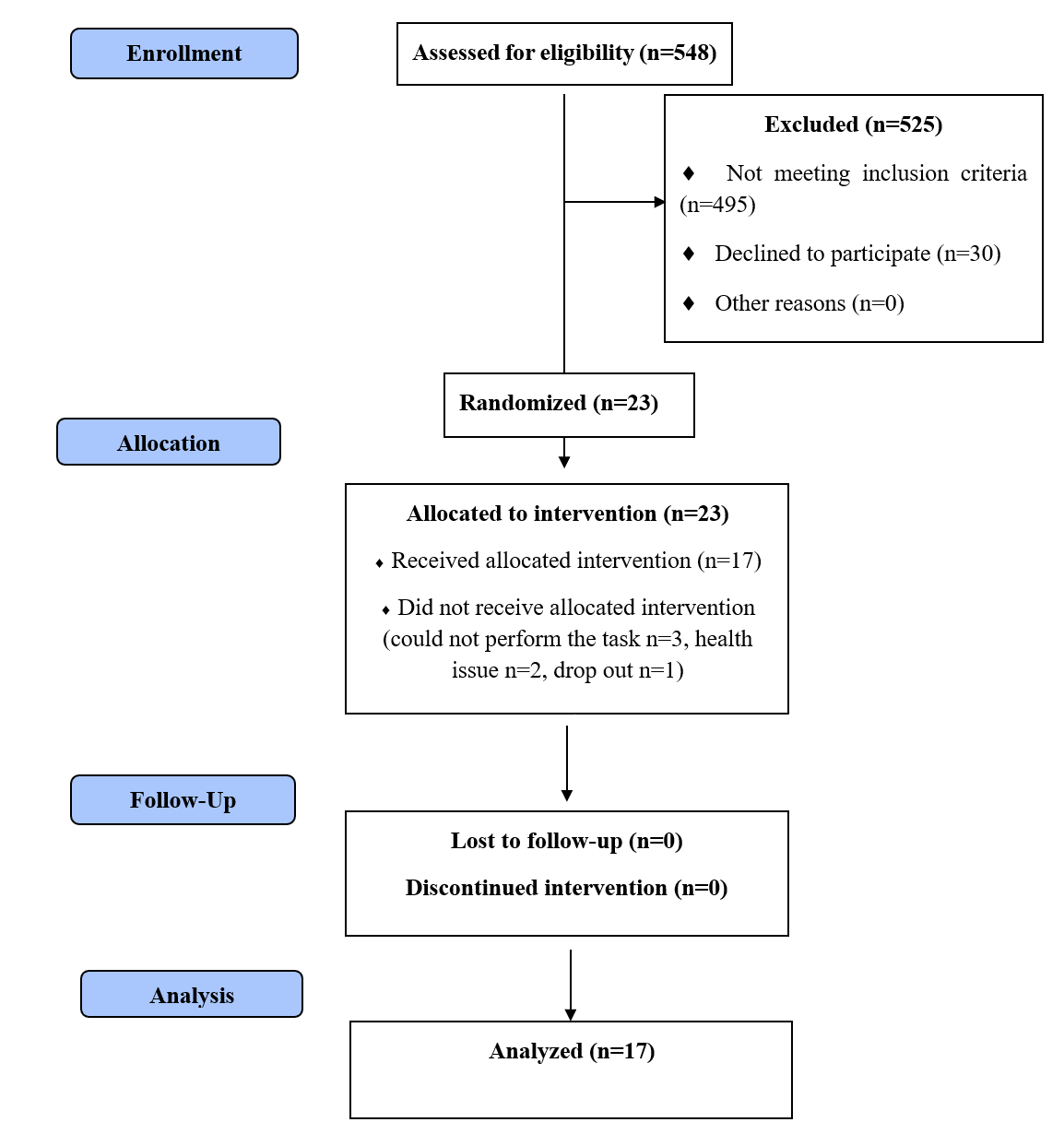


The CONSORT flow diagram has been simplified because the RCT assumed a crossover design. Three factors were randomized and balanced across patients: (1) tDCS condition (real/sham), (2) bimanual configuration (i.e., the direction of movements controlled by the paretic upper limb: lateral or sagittal), and (3) CIRCUIT’s version (versions 1-4: same length and difficulty but different segment order).

Supplementary Figure 2. Imaging of stroke patients


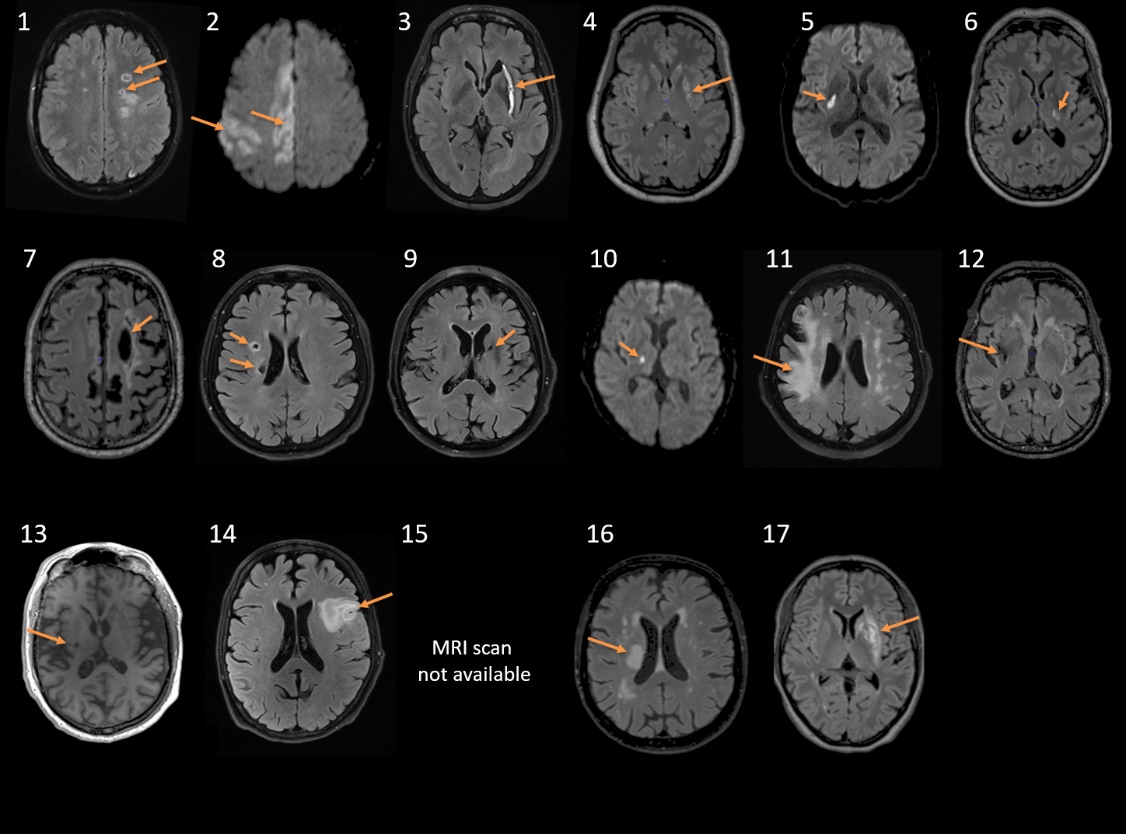


MRI scanners of chronic stroke patients. For Patient #15, only the radiological report (citing acute ischemic stroke in the territory of the anterior choroidal artery involving the posterior limb of the internal capsule) was available.

# Supplementary Methods

## Randomization

The randomization was performed by a third person using the online minimization software QMin® (http://rct.mui.ac.ir/q/index.php), which provided the tDCS codes and the bimanual configuration. The randomization criteria were as follows: gender (M/F), age (18-50/50-70 years), time since stroke (6-12 months/1-3 years/>3 years), stroke laterality (dominant/nondominant hemisphere), upper limb deficit severity ((near-)normal hand/useful hand/nonfunctional hand/minimal arm movements), stroke localization (cortical/subcortical), prior exposure to tDCS (yes/no), and prior use of REAplan® (yes/no).

**REAplan® robot and serious games**

The instructions were provided. Virtual walls were imposed by the REAplan®. One hand controlled exclusively lateral (left-right, X-axis) displacements of the common cursor displayed on the screen. The other hand controlled exclusively sagittal (forward-backward, Y-axis) movements. The cursor’s X-Y positions, velocities and forces exerted against the virtual walls were sampled at 80 Hz and stored for off-line analysis.

# Supplementary Results

Supplementary Figure 3. Absence of carry-over effect

Evolution for biSAT on the bimanual CIRCUIT in stroke patient. Lack of a carry-over effect of the order of time, tDCS condition, bimanual configuration, or of the [time × stimulation order × bimanual configuration] interaction. RS: Real then Sham, SR: Sham then Real.

Supplementary Figure 4. Bimanual improvement in a stroke patient


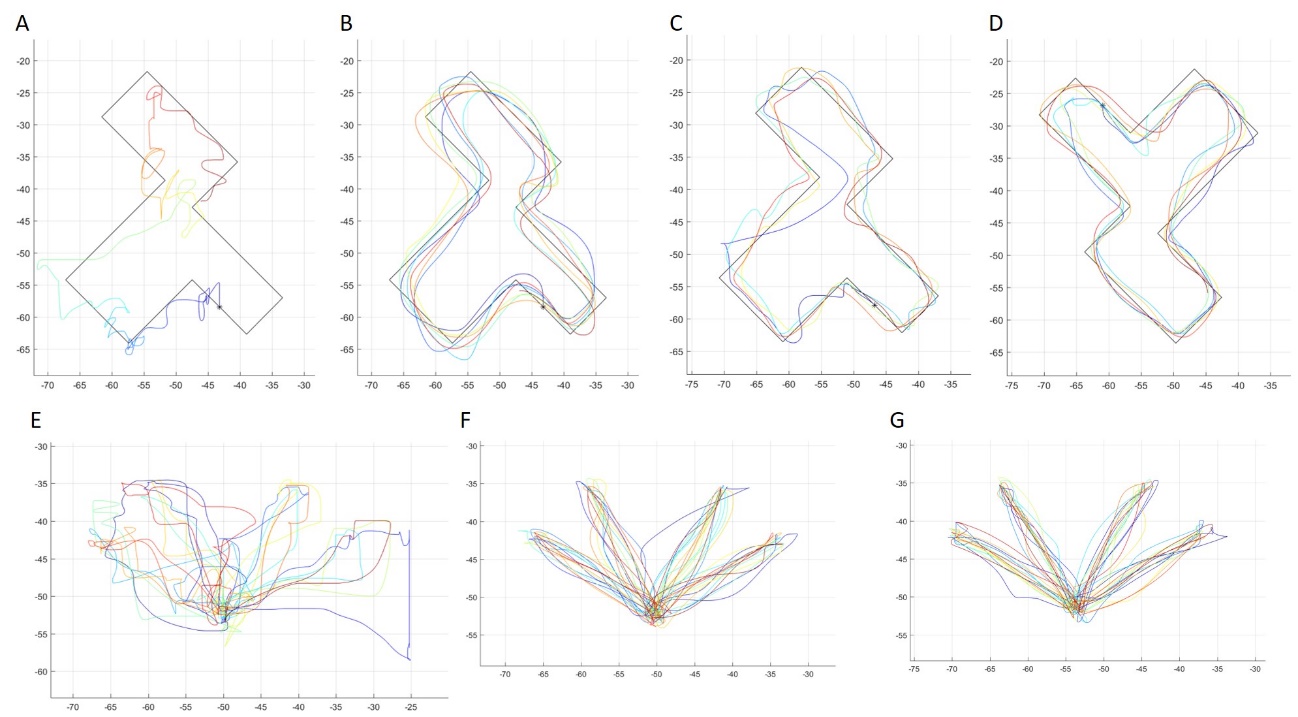


Upper row. Performance on the bimanual CIRCUIT task during the following blocks: A: Baseline, B: 60 min after the end of training and tDCS, C: retention 1 (R1) one week after intervention, and D: generalization (G) on a new circuit.

Lower row. Performance on the bimanual REACHING task during the following blocks: E: Baseline, F: 60 min after the end of training and tDCS, and G: retention 1 (R1) one week after intervention.

Supplementary Figure 5. Healthy individuals and stroke patients


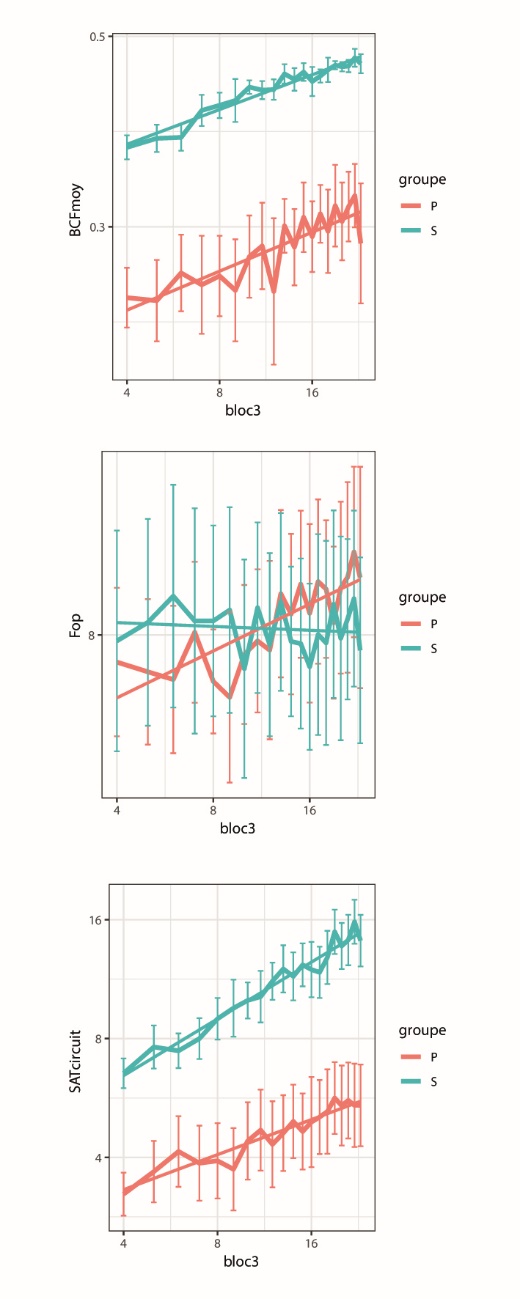


Evolution of biSAT, biCO and biFOP in the 7 healthy individuals (no tDCS) and the 17 stroke patients during their sham session, mean ±SD. S: healthy individuals, P: patients.
